# Type 2 diabetes, breast cancer specific and overall mortality: Associations by metformin use and modification by race, body mass, and estrogen receptor status

**DOI:** 10.1371/journal.pone.0232581

**Published:** 2020-05-05

**Authors:** Kyung Na Lee, Mylin A. Torres, Alyssa N. Troeschel, Jiabei He, Keerthi Gogineni, Lauren E. McCullough

**Affiliations:** 1 Department of Epidemiology, Emory University, Atlanta, Georgia, United States of America; 2 Department of Radiation Oncology, Emory University, Atlanta, Georgia, United States of America; 3 Glenn Family Breast Center of the Winship Cancer Institute, Emory University, Atlanta, Georgia, United States of America; 4 Department of Medical Oncology, Emory University, Atlanta, Georgia, United States of America; Johns Hopkins University School of Medicine, UNITED STATES

## Abstract

**Introduction:**

While type 2 diabetes (T2D) has been associated with increased all-cause mortality among women diagnosed with breast cancer (BC), the association between T2D and breast cancer-specific (BCS) mortality is unresolved. The goal of this study was to examine the association between T2D and BCS mortality and examine the influence of metformin treatment on mortality rates.

**Methods:**

A retrospective cohort study was conducted between 2002 and 2008 at Emory University Hospitals among non-Hispanic black and white women who had confirmed diagnosis of stage I-III BC and known diabetes status (T2D: n = 73; non-T2D: n = 514). Cox proportional hazard models were used to estimate hazard ratios (HR) and 95% confidence intervals (CI).

**Results:**

Compared to non-T2D patients, T2D women had almost a 2-fold increase in BCS mortality after adjusting for covariates (HR = 2.01; 95%CI = 1.02–3.98). Though attenuated, the increased hazard of death was also observed for all-cause mortality (HR = 1.74; 95%CI = 1.06–2.87). T2D patients who were not on metformin had substantially higher hazard of BCS mortality compared to non-diabetic patients (HR = 4.54; 95%CI = 1.98–10.44), whereas the association among T2D patients treated with metformin was weak (HR = 1.20; 95%CI = 0.36–3.97) and included the null.

**Conclusions:**

Among women with BC, T2D is associated with increased BCS mortality. Metformin treatment for T2D during the initial diagnosis of BC may improve outcomes.

## Introduction

Breast cancer is the leading cause of non-cutaneous cancer-related illness among women, and the second leading cause of cancer-related death in the United States (U.S.) [1]. Although the incidence of breast cancer among non-Hispanic white women is slightly higher than non-Hispanic black women (128.1 versus 124.3 cases per 100,000), non-Hispanic blacks are approximately 42% more likely to die of breast cancer than white counterparts [1].

Obesity is an established risk factor for postmenopausal breast cancer and a prognostic factor for all women (irrespective of age). Obesity, combined with inadequate diet and exercise, increases insulin resistance and hyperinsulinemia and is a risk factor for type 2 diabetes (T2D). T2D has not only been implicated in the etiology of postmenopausal breast cancer [1–5], but is related to poor survival [6–13] and is independently associated with breast cancer cell proliferation, invasiveness, angiogenesis, and reduced cell apoptosis [14–16]. Despite the body of evidence supporting the association between T2D and overall mortality [6–13], the association between T2D and breast cancer-specific (BCS) mortality is unresolved—with some studies supporting increased mortality rates [7, 13, 17–19], while others show no increased risk of BCS mortality [20–22]. In addition, the influence of important breast cancer prognostic factors (e.g., body mass index [BMI kg/m^2^] and estrogen receptor [ER] status) on the relationship between T2D and breast cancer outcomes are unclear [23–27]. Further, it is suggested that the association between T2D and breast cancer mortality may be modified by race with black diabetic women being more likely to die of breast cancer than their Caucasian counterparts [23, 26, 28] and could inform our understanding of racial disparities in breast cancer mortality.

The biological mechanisms linking T2D and breast cancer progression may be related to insulin resistance and activation of insulin receptor, or inhibition of the adenosine monophosphate-activated protein kinase (AMPK) pathway in breast tissue [14,15]. Thus, understanding the association between T2D and breast cancer mortality in the context of diabetic medications may be crucial to improving outcomes in this population [29–31]. Metformin is a first-line therapy for treatment of T2D and has shown in numerous studies to produce anti-cancer effects through inhibition of the mammalian target of rapamycin (mTOR) pathway, which is important in controlling cancer cell proliferations and cell growth [32–34]. Although metformin’s potential to improve BCS survival among T2D patients is promising, previous studies of diabetic medications and mortality in breast cancer patients have been inconclusive, with some finding improved survival [6, 30–33, 35–42], and others showing no difference with diabetic medication treatment [43–46].

We retrospectively reviewed the charts of a racially diverse cohort of women treated for breast cancer at Emory University Hospital in Atlanta, GA to examine the relationship between T2D and mortality, as well as the association by T2D medication use (metformin vs. no metformin). Given the diversity of patients by race, BMI and ER status in the metro-Atlanta area, we additionally explored the modifying effects of these factors on the relationship betweenT2D and mortality following a diagnosis of breast cancer.

## Methods

### Study population

We included 608 women from the radiation oncology database at Emory University who had confirmed diagnosis of stage I-III invasive breast cancer (ICD: C509) and a biopsy or surgical date between January 1, 2002 and December 31, 2008. After review of the electronic medical record (EMR), we excluded 21 women with unknown diabetes status, type 1 diabetes, gestational diabetes, and patients diagnosed with diabetes after breast cancer diagnosis. A total of 587 women (non-Hispanic white N = 357, non-Hispanic black N = 230) were included in our final analytic dataset. Ethics approval and waiver of informed consent for the study was granted by the Institutional Review Board at Emory University (IRB00018512).

### Exposure assessment

All subjects had biopsy proven breast cancer. Patient’s T2D status and medications for T2D were recorded from the EMR and based on concurrent disease at the time of breast cancer diagnosis. In our cohort, 73 patients (N = 27 non-Hispanic white, N = 46 non-Hispanic Black) had T2D. Diabetic women were further divided based on their diabetic medications prescribed at the time of breast cancer diagnosis: 31 women were on metformin as either mono or combination therapy (metformin users); 27 women were on either sulfonylureas, thiazolidiones, or insulin as mono or combination therapy (non-metformin users); and 15 diabetic women received lifestyle modifications only (no medication users). Among the “no medication users”, only one event (death) was observed. As a result, this group was combined with non-metformin users for all analyses. Following the intent to treat approach, patients were divided into each group based on diabetic status and medications that they were prescribed at the time of breast cancer diagnosis.

### Outcome assessment

Two separate outcomes of interest, BCS mortality and overall mortality, were obtained from the Georgia Cancer Registry that links to both the State and National Death Index. Follow-up was considered complete through November 3, 2016. BCS mortality was defined as the time from diagnosis until death due to the breast cancer, and overall mortality was defined as the time from diagnosis until death from any cause (including, but not limited to breast cancer). Women accrued time from the date of diagnosis until either death from any cause or the end of the follow-up period (November 3, 2016).

### Covariate assessment

The following patient-related factors, obtained at diagnosis, were included in models as potential effect measure modifiers or covariates: age at breast cancer diagnosis (continuous); race (non-Hispanic white vs. non-Hispanic black); obesity (BMI kg/m^2^<30 vs. BMI kg/m^2^≥30); menopausal status (pre-menopausal vs. post-menopausal); and comorbid conditions (hypertension, heart disease, hypercholesterolemia, and other disease). In addition, the following clinical characteristics were evaluated: American Joint Committee on Cancer stage (7^th^ edition) (stage I, II, and III) [47]; tumor grade (grade I/II vs. III) and ER status (positive vs. negative).

### Statistical analysis

All statistical analyses were performed using SAS 9.4 (SAS Institute Inc., Cary, NC). Patient demographics and clinical characteristics were compared using chi-square and Fisher’s exact tests. The association between T2D and BCS or overall mortality was evaluated by generating multivariable adjusted survival curves. Cox proportional hazards regression analysis [48] was conducted to estimate the associations of T2D and metformin use with mortality following breast cancer diagnosis. We performed visual assessment of the proportional hazards assumption in addition to goodness of fit tests. No violations were observed. Potential modification of the association between T2D and mortality was evaluated by race, BMI, and ER status using the likelihood ratio test (LRT) to assess interaction on the multiplicative scale. We additionally assessed interaction on the additive scale using the interaction contrast ratio (ICR) test [49]. Due to limited number of breast cancer deaths (N = 75), we restricted these analyses to overall mortality comparing T2D vs. non-T2D in strata of race, BMI, and ER status.

All potential confounders were identified based on a directed acyclic graph (DAG) [48]. Clinical characteristics were removed from all models as they were on the causal pathway between T2D and mortality, as the presence of T2D preceded the cancer diagnosis and is posited to associate with clinical factors such as stage and subtype given shared risk factors [*e*.*g*., obesity]). Inclusion of these characteristics would thus violate our confounding criteria. Our final models included age at diagnosis, race, BMI, and comorbid conditions.

## Results

### Patient demographics and clinical characteristics

Table 1 represents patient demographics and clinical characteristics, categorized by patient’s T2D status. In comparison to non-T2D, T2D women were more likely to be older, Non-Hispanic black, obese, post-menopausal at breast cancer diagnosis, and present with more comorbid conditions. With the exception of tumor grade, the two groups were similar with respect to clinical characteristics.

**Table 1:** Patient demographics and clinical characteristics at time of breast cancer diagnosis, by T2D status.

Characteristics	T2D (N = 73)	Non-T2D (N = 514)	P-value
Age at diagnosis, years			< .0001
Median (range)	62.00 (32–82)	52.50 (22–89)	
	No. (%)	No. (%)	
Race			< .0001
Non-Hispanic white	27 (36.99)	330 (64.20)	
Non-Hispanic black	46 (63.01)	184 (35.80)	
BMI (kg/m^2^)			0.0003
< 30 (non-obese)	41 (56.16)	360 (70.04)	
≥ 30 (obese)	28 (38.36)	125 (24.32)	
Missing	4 (5.48)	29 (5.64)	
Menopausal status			< .0001
Pre-menopausal	5 (6.85)	194 (37.74)	
Post-menopausal	65 (89.04)	300 (58.37)	
Missing	3 (4.11)	20 (3.89)	
Comorbidities			< .0001
No	7 (9.59)	162 (31.52)	
Yes (≥1 condition)	66 (90.41)	348 (67.70)	
Hypertension	53 (72.60)	180 (35.02)	
Heart Disease ^a^	17 (23.29)	40 (7.78)	
Hypercholesterolemia	27 (36.99)	66 (12.84)	
Others	10 (13.70)	180 (35.02)	
Missing	0 (0.00)	4 (0.78)	
**Clinical Characteristics**			
Clinical stage			0.1028
I	28 (38.36)	198 (38.52)	
II	22 (30.14)	209 (40.66)	
III	23 (31.51)	107 (20.82)	
Tumor grade			0.0327
I and II	33 (45.21)	283 (55.06)	
III	33 (45.21)	182 (35.41)	
Missing	7 (9.59)	49 (9.53)	
ER Status			0.1373
positive	46 (63.01)	342 (66.54)	
negative	23 (31.51)	162 (31.52)	
Missing	4 (5.48)	10 (1.95)	

T2D type 2 diabetes, BC breast cancer, BMI body mass index, ER estrogen receptor

^a^ Includes arterial fibrillation, arrhythmia, coronary artery disease, cardiovascular disease, myocardial infarction, and mitral valve prolapse

### BCS and overall mortality rates by T2D status and metformin use

**Fig 1:**
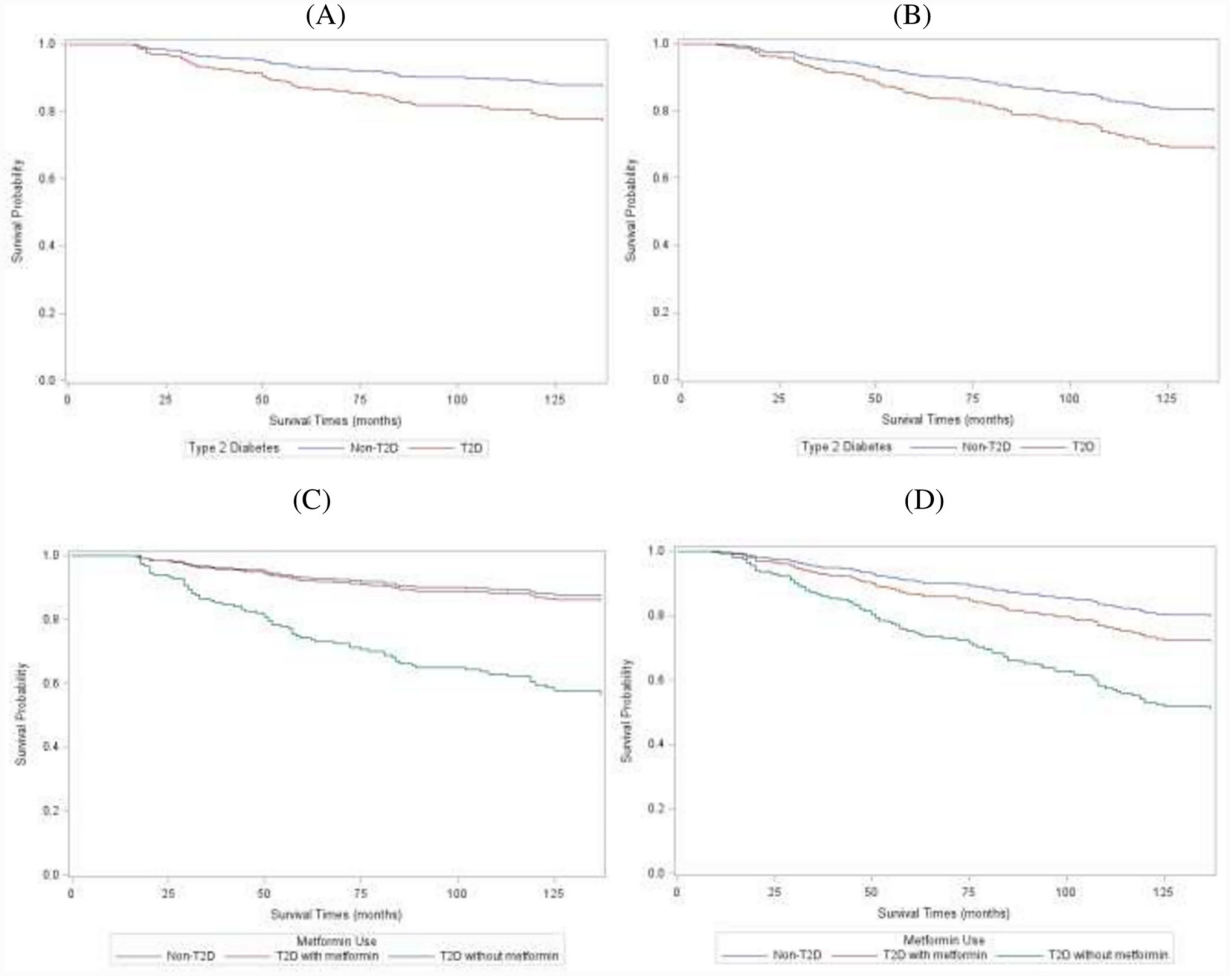
Multi-variable adjusted survival curves for breast cancer specific survival (BCSS) by T2D status (a), overall survival (OS) by T2D status (b), BCSS by T2D status and metformin use (c), and OS by T2D status and metformin use (d).

With a mean follow-up time of 93.26 months (range, 5–173 months) among T2D women, 25 (34.2%) deaths occurred, 13 (17.8%) due to breast cancer. In the non-T2D group, the mean follow-up time was 115.10 months (range, 1–177 months). During this time 98 (19.1%) deaths occurred, in which 62 (12.1%) deaths were due to breast cancer. Fig 1 shows multi-variable adjusted survival curves for breast cancer specific survival (BCSS) (Fig 1a) and overall survival (OS) (Fig 1b) by T2D status. Compared to non-T2D, T2D women had a 2-fold increase in BCS mortality (HR = 2.01; 95% = CI 1.02–3.98) after adjusting for age at diagnosis, race, BMI, and comorbidities (Table 2). The risk of death attenuated with overall mortality (HR = 1.74; 95% CI = 1.06–2.87) after adjusting for the same covariates. Our multi-variable survival curves for BCCS and OS show improved survival among T2D women with metformin compared to T2D women without metformin (Fig 1c and 1d). Although the risk of BCS mortality among T2D patients on metformin was slightly higher than non-T2D patients (HR = 1.20; 95% CI = 0.36–3.97), these women had less risk of BC death than T2D patients not on metformin (HR = 4.54; 95% CI = 1.98–10.44). Similar trends were observed for overall mortality where slightly higher death rate was shown among metformin users compared to women without T2D (HR = 1.51; 95% CI = 0.74–3.10), but non-metformin users had a more than 3-fold risk of death compared to women without T2D (HR = 3.21; 95% CI = 1.63–6.31).

**Table 2:** Multivariable Cox proportional hazard model of the association between T2D status and mortality, overall and by diabetes medication.

	BCS Mortality			Overall Mortality		
	events/ p-month^a^	HR^b^	95% CI	events/ p-month^a^	HR^b^	95% CI
**Main effect**						
non-T2D	62/55,824	1.00	Ref	98/55,824	1.00	Ref
T2D	13/6,062	2.01	1.02–3.98	25/6,062	1.74	1.06–2.87
**Diabetes Medication**						
non-T2D	62/55,824	1.00	Ref	98/55,824	1.00	Ref
T2D with metformin	4/3,082	1.20	0.36–3.97	10/3,082	1.51	0.74–3.10
T2D with no metformin	9/1,664	4.54	1.98–10.44	15/1,664	3.21	1.63–6.31

### Overall mortality rates by T2D status within strata of race, BMI, and ER status

#### Race

Among Non-Hispanic whites, overall survival rates were slightly higher for T2D than non-T2D (HR = 1.05; 95% CI = 0.41–2.73). However, among non-Hispanic blacks, a greater than 2-fold increase in overall mortality was observed in T2D patients compared to non-T2D women (HR = 2.44; 95% CI = 1.25–4.77). Using single referent coding to estimate the joint probability of risk, non-Hispanic blacks with T2D had the worst BCS mortality rates (HR = 3.04; 95% CI = 0.54–6.01) (Table 3). We found no significant interactions by race on either multiplicative or additive scales.

**Table 3:** Multivariable Cox proportional hazard model of the association between T2D status and overall mortality by race, BMI and ER status.

	Overall Mortality				
		Stratified HR^b^		Single referent HR^c^	
	events/ p-month^a^	HR (95% CI)	LRT P-value	HR (95% CI)	Additive ICR/RERI (95% CI)
**Race**			0.13		
Non-Hispanic White					
non-T2D	52/37,014	1.00		1.00	
T2D	5/2,801	1.05 (0.41–2.73)		1.05 (0.41–2.73)	
Non-Hispanic Black					
non-T2D	46/18,810	1.00		1.24 (0.76–2.05)	
T2D	20/3,261	2.44 (1.25–4.77)		3.04 (0.54–6.01)	1.74 (-0.34–3.82)
**BMI (kg/m**^**2**^**)**			0.18		
< 30 (non-obese)					
non-T2D	64/40,314	1.00		1.00	
T2D	10/3,254	1.18 (0.49–2.83)		1.18 (0.49–2.83)	
≥ 30 (Obese)					
non-T2D	24/13,305	1.00		1.21 (0.72–2.02)	
T2D	13/2,551	2.44 (1.20–4.95)		2.95 (1.55–5.61)	1.57 (-0.41–3.54)
**ER Status**			0.88		
ER positive					
non-T2D	49/38,123	1.00		1.00	
T2D	13/4,136	1.72 (0.87–3.41)		1.72 (0.87–3.41)	
ER negative					
non-T2D	45/16,988	1.00		2.19 (1.40–3.44)	
T2D	9/1,775	1.86 (0.80–4.35)		4.08 (1.76–9.49)	1.17 (-0.67–3.00)

T2D type 2 diabetes, BMI body max index, ER estrogen receptor, HR hazard ratios, CI confidence intervals, LRT likelihood ratio test, ICR/RERI interaction contrast ratio/relative excess risk due to interaction

^a^ events/p-month: number of death occurred by the sum of total time in months contributed by all patients

^b^ The effect of T2D status on overall mortality on multiplicative scale

^c^ Combined effect of T2D status and 3 different effect measure modifiers on additive scale

#### BMI

Among obese women with T2D we observed a greater than 2-fold increase in overall deaths compared to non-T2D obese women (HR = 2.44; 95% CI = 1.20–4.95). Using the single referent approach, we similarly found that obese women with T2D were at a near 3-fold increased risk of overall mortality (HR = 2.95; 95% CI = 1.55–5.61), followed by non-T2D obese women (HR = 1.21; 95% CI = 0.72–2.02) (Table 3), although interactions on multiplicative or additive scales were not statistically significant.

### ER status

In stratified analyses, we observed no notable difference in the hazard of death among ER positive breast cancer patients with T2D and ER negative breast cancer cases with T2D. When considering the estimated HR for the joint effect of T2D and ER status, a 4-fold increase in hazard was observed among women jointly classified with T2D and ER negative breast cancer (HR = 4.08; 95% CI = 1.76–9.49) (Table 3). Interactions between T2D and ER status were not present on multiplicative or additive scales.

## Discussion

In our study, breast cancer patients with T2D had a two-fold increased risk of BCS mortality and 74% increased overall mortality compared to breast cancer patients without T2D. Mortality rates were highest among women with T2D who were not receiving metformin at the time of breast cancer diagnosis. Our findings may suggest that having T2D is a strong prognostic factor for breast cancer-related mortality, and that metformin treatment for T2D during the initial diagnosis of BC may improve outcomes. Although we did not observe statistically significant interactions between T2D and race, BMI, or ER status on multiplicative or additive scales, the association of T2D with all-cause mortality appeared stronger among non-Hispanic blacks and among those with a BMI ≥ 30 kg/m^2^. Lack of statistically significant interactions is likely due to limited power for stratified analyses, but the results suggest assessing effect measure modification (EMM) by these factors may be important for targeting future interventions.

The positive association between T2D status and overall mortality observed in our study is consistent with a meta-analysis where a 37% increased risk of overall mortality was observed (HR = 1.37; 95% CI = 1.34–1.41) [18]. This result was in agreement with other observational studies as well [6, 17, 19, 32, 50–52]. However, previous studies on the association between T2D and BCS mortality rates are mixed. Two prospective studies conducted by Yeh et al., and Chen et al. [19, 53] (n = 18,280 and n = 4,390 respectively) showed 27% and 53% increased BCS mortality (95% CI = 0.17–9.73 and 95% CI = 1.14–2.05 respectively), which was further supported by a retrospective cohort study among Swedish women diagnosed with breast cancer (n = 146,764) where the hazard of BCS death was 1.45 (95% CI = 1.32–1.59) [13].

In contrast, two retrospective studies with relatively large sample size showed no associations between T2D status and BCS mortality rates [20, 22]. In a prospective cohort (n = 4,664) by Nechuta et al., investigators identified no significant association (HR = 0.98; 95% CI = 0.68–1.41) [22], consistent with finding from Luo et al. (n = 8,108) after accounting for competing risks of death (HR = 0.90; 95% CI = 0.65–1.24) [20]. Despite the conflicting results, there is a general consensus that T2D at the time of breast cancer diagnosis is associated with mortality among breast cancer patients [7, 13, 17–19, 28, 34, 53]. Our result of a 101% increase in BCS mortality is consistent with this, although, given the relatively small sample size and limited number of patients who experienced a breast cancer–related death (n = 13), should be interpreted with caution.

Increased risk of death among T2D patients with breast cancer is thought to be both directly and indirectly related to hormonal, inflammatory, or metabolic characteristics of T2D [18]. T2D, often combined with obesity, induces hormonal changes by increasing insulin resistance, inflammation with increased Interleukin 6, and increased reactive oxygen species, all of which can lead to hyperinsulinemia and insulin-stimulated mitosis by directly affecting cancer cell proliferation [54]. Increased insulin level can also indirectly stimulate tumor cell growth by increasing the bioavailability of insulin-like-growth-factor-1 (IGF-I) through glucogenesis, which can activate insulin receptors in various tissues including breast cells through stimulation of abnormal signaling cascade that can increase angiogenesis and reduced apoptosis [16,54]. In addition, hyperinsulinemia can decrease sex-hormone-binding globulin that can increase estrogen concentration and enhance estrogen receptor activation through IGF-I signaling [54]. It is known that a synergistic effect of estrogen and IGF-I can lead to breast cancer cell proliferation among diabetic patients [16].

Given the potential mechanisms linking T2D and breast cancer mortality, diabetic medications such as metformin may have a favorable effect on survival among breast cancer patients. Both in vitro studies and observational clinical studies support metformin’s anti-cancer benefit as compared to other diabetic medications. It is thought to have therapeutic effect as a mono-therapy, or synergistic effect when combined with chemotherapeutic drugs, to suppress cancer cell proliferation and increase apoptosis in breast cancer cells [55]. Hyperinsulinemia has been consistently associated with increased breast cancer risk, and metformin is known to provide both direct and indirect effect in reducing cell proliferation through controlling insulin levels and blood glucose [34]. Metformin indirectly activates AMPK, which then inhibits the mTOR to prevent breast cancer cell proliferation as well as to stop cell growth and pathological cell cycle progression [34].

Previous studies on the association between metformin and breast cancer outcomes have mixed results, although growing evidence suggests its potential effect as an anti-cancer agent. A retrospective clinical meta-analysis of 28 studies conducted among breast cancer patients with concurrent diabetes showed an inverse association between metformin and overall mortality with a pooled effect estimate of 0.70 (95% CI = 0.55–0.88) [56]. Moreover, a Danish population-based study (n = 1,058) showed reduction in both BCS and overall mortality with cumulative metformin use (HR = 0.74; 95% CI = 0.58–0.96 and HR = 0.88; 95% CI = 0.59–1.29 respectively) [40]. Although our study result was based on relatively small number of events, and effects imprecise, the observed hazard of death was considerably lower among metformin users than non-metformin users when compared to women without T2D, which may suggest that metformin could attenuate the deleterious effects of T2D on mortality following a diagnosis of breast cancer.

In our study, we additionally explored EMM by strata of race, BMI, and ER status. Although EMM on two different scales was not statistically significant, additive interaction was in the positive direction, suggesting synergy between the covariates of interest and T2D status. Obese patients with T2D may have delayed detection due to increased breast fat tissue, altered biological mechanisms due to macrophage infiltration, more aggressive breast tumors, and increased odds of comorbid conditions [27,57]. Moreover, at least one previous study has shown increased risk of ER negative breast cancer among non-Hispanic blacks with T2D [23], suggesting that T2D control in this group may narrow mortality disparities. While our data were suggestive, and biologically plausible, larger samples with incident T2D cases are needed to make strong conclusions on these potential interactions.

The strengths of our study include a diverse cohort of women diagnosed with stage I to III invasive breast cancer and our statistical approach, which assessed both multiplicative and additive effects. Accessing additive interaction may be better reflect biologic interaction [48] and be more relevant for public health intervention. A major limitation of our study is that participants were from a highly selected pool of patients, and thus our study results may not be externally valid. Using the intent to treat approach, diabetes status was based on T2D status at the time of breast cancer diagnosis. We were unable to capture changes in therapy over time or adherence to medications as prescribed; potentially leading to misclassification. We also did not take into consideration T2D severity, duration of T2D therapy, or have sufficient power to examine treatment regimens, all of which may affect breast cancer progression independent of the anti-proliferative effect of metformin. Similarly, we were unable to understand the effects of metformin independent of other therapies, as patient level details of combination therapies were not available. Finally, the number of women with T2D and those who experienced an event were relatively small. Thus, we were underpowered to detect differences by key covariates on both multiplicative and additive scales, and similarly unable to report medication-specific findings in strata of race.

## Conclusions

Our findings show that prevalent T2D status at the time of breast cancer diagnosis can increase BCS mortality and all-cause mortality among the women diagnosed with stage I-III invasive breast cancer. Further study is needed to investigate the causal relationship between T2D and breast cancer in clinical settings, particularly in subgroups of race and BMI, where targeted intervention (such as metformin) may attenuate the excess risk of deaths among breast cancer patients with T2D.
